# Association between tinnitus and cognitive impairment: analysis of National Health and Nutrition Examination Survey 2011:2014

**DOI:** 10.3389/fneur.2025.1533821

**Published:** 2025-05-30

**Authors:** Jianli Wu, Mengdi Shi, Chao Wang

**Affiliations:** ^1^Institute of Traditional Chinese Medicine, Heilongjiang University of Chinese Medicine, Harbin, China; ^2^Graduate School of Heilongjiang University of Traditional Chinese Medicine, Harbin, China

**Keywords:** cognitive impairment, cognitive testing, dementia, tinnitus, NHANES

## Abstract

**Background:**

Tinnitus is one of the most common potential risk factors for cognitive impairment. To understand the relationship between tinnitus and cognitive impairment, the National Health and Nutrition Examination Survey (NHANES) database was analyzed after adjusting for potential confounders, including age and other systemic comorbidities.

**Materials and methods:**

A total of 684 participants who had undergone a total tinnitus survey and underwent a cognitive function test were included. Tinnitus is divided into acute tinnitus and non-acute tinnitus. The Consortium for the Establishment of Alzheimer’s Disease Word Learning Xi Registry (CERAD-WL), the Dynamic Logistics Proficiency Test (AFT), and the Digit Sign Substitution Test (DSST) were used to assess cognitive impairment.

**Results:**

Subjects with tinnitus had lower AFT and DSST scores compared to the healthy group, indicating decreased cognitive function. After adjusting for other covariates in humans, tinnitus was significantly associated with a decrease in AFT and DSST scores (trend *p*-values = 0.02 and 0.005, respectively).

**Conclusion:**

Tinnitus is associated with cognitive impairment.

## Introduction

With the increasingly serious problem of population aging, more and more countries in the world are facing a series of problems caused by population aging In recent years, the number of elderly people with cognitive impairments has been rapidly increasing, making cognitive impairment the most prominent global issue among the elderly population (1, 2). Cognitive impairment has a great influence on the excellent of existence of the aged, while also imposing a substantial burden on the socioeconomy (3). The best current approach to cognitive impairment is to identify modifiable risk factors, which may slow down the progression of cognitive decline associated with aging, and prevent or delay the occurrence of cognitive impairment or dementia (3). Age is currently the primary risk factor for cognitive impairment, but there are also many other diseases that can lead to a decline in cognitive ability (4).

Patients with tinnitus report an unspecified auditory sound, such as ringing, but there are also buzzing, clicking, pulsating sounds, and other noises.

Tinnitus is viewed a symptom of an underlying situation as an alternative than a disease, referring to the appreciation of sound in the head or ears except corresponding exterior sound (5). Additionally, studies have indicated that tinnitus is an age-related condition (6), Furthermore, research has shown that tinnitus has certain risk factors similar to cognitive impairment, such as hypertension, diabetes, stroke (7, 8). Moreover, research suggests that tinnitus or hearing loss may be related to the onset rate of cognitive impairment (9). Furthermore, some scholars have proposed that tinnitus impairs “executive attention” leading to poorer cognitive abilities (10, 11). Some also believe that changes in the brain’s structure and function in tinnitus patients are associated with cognitive impairment. Tinnitus can lead to enhanced functional connectivity in the posterior cingulate cortex and abnormal late auditory evoked potentials (12).

Currently, there is limited research to prove the relationship between tinnitus and cognitive impairment. Therefore, the motive of this learn about is to analyze the affiliation between tinnitus and cognitive impairment.

Emerging evidence suggests that tinnitus may also contribute to cognitive deficits, particularly in domains such as attention, memory, and executive function few studies have rigorously disentangled the effects of tinnitus duration—acute versus non-acute—on cognitive outcomes, despite theoretical models positing that prolonged tinnitus may exacerbate neural resource depletion through maladaptive neuroplasticity. However, the nature of this association remains poorly understood, with extant literature marked by conflicting findings and methodological heterogeneity.

## Materials and methods

### Data sources

The data used in this study is derived from NHANES, a stratified, multistage probability cluster survey conducted among non-institutionalized civilians in the United States (13). The survey includes household interviews and physical examinations conducted in Mobile Examination Centers (MEC). Ethical approval was once bought from the Institutional Review Board of the National Center for Health Statistics, and all survey individuals furnished written knowledgeable consent. We analyzed a population of individuals aged 60 and above who fully completed cognitive function tests and tinnitus surveys. These individuals also completed examinations for covariates such as gender, BMI, smoking status, and alcohol consumption (*n* = 684).

The NHANES survey was approved by the institutional review board of the US Centers for Disease Control and Prevention (CDC). Informed consent was obtained from all sampled persons (for those aged <18 years, assent with proxy consent). All data were de-identified and made publicly available. More detailed descriptions of the NHANES survey are available on the CDC website.

### Tinnitus

Considering clinical practice guidelines and the characteristics of the NHANES database, tinnitus can be categorized into three groups: normal participants, patients with acute tinnitus, and patients with non-acute tinnitus. Participants are required to answer the following questions: “How long been bothered by this ringing, roaring, or buzzing in ears or head?” Participants who answered “none” will be categorized as normal participants, those who answered “less than 3 months” will be categorized as having acute tinnitus, and the rest of the participants will be categorized as having non-acute tinnitus (14, 15).

### Cognitive impairment

NHANES utilizes the following approaches to evaluate memory function: establishment of the Consortium to Establish a Registry for Alzheimer’s Disease Word List (CERAD-WL), Animal Fluency Test (AFT), and Digit Symbol Substitution Test (DSST). Cognitive abilities, encompassing episodic memory, semantic memory, and working memory/executive function, are assessed through questionnaires.

CERAD-WL is a reliable and valid cognitive function assessment scale consisting of three learning trials, one of which is a delayed trial. Participants are instructed to audibly read 10 randomly selected and unrelated words. After presenting these words visually or auditorily to the participants, the words are displayed one by one on a monitor, followed by an immediate recall of as many words as possible. The whole rating for immediately mastering and recall is calculated by way of summing the quantity of right solutions from every participant in every spherical (range: 0–30). The delayed trial begins round 8–10 min after the first spherical of on the spot recall (typically carried out after finishing AFT and DSST tests). Participants are requested to recall as many of these 10 phrases as they can. The CERAD-Total rating is calculated by using summing the rankings of the three instant trials and one delayed trial, with a most rating of 40 (16).

The Animal Fluency Test is a easy technique to measure semantic fluency. This test is frequently employed in clinical and research contexts. Patients are instructed to produce as many animal names as they can within a one-minute timeframe. One point is given for each animal listed (16).

The DSST serves as a worldwide metric for assessing brain health. It is primarily based on processing speed, visible scanning, sustained attention, and non-permanent reminiscence (17). Because of its simplicity and outstanding discriminant validity, it has become one of the most frequently used tests in current clinical neuropsychology. It is utilized for the comparison of working memory, processing speed, and sustained interest.

The better the cognitive abilities, the higher the scores in all three tests. At present, there is no definitive cutoff threshold for defining cognitive impairment, but previous research has indicated that individuals with the lowest scores have cognitive impairments.

In summary, this study will follow previous research in using DSST scores as the criterion for assessing cognitive impairment. The study population is defined as having cognitive impairment or cognitive dysfunction based on the unweighted lower quartile (DSST ≤ 36). Participants who score above 36 on the DSST are regarded as not having cognitive impairment (18).

### Covariates

In addition, this study gathered other covariates that have been identified in the published literature as potential risk factors for cognitive impairment, including gender, race, income, education level, alcohol consumption, diabetes. Dopamine (DA), a neurotransmitter essentially known for its relevance in the regulation of behavior and movement, modulates cognitive function, too. Interestingly, alterations of the dopaminergic system have been observed in DM. So we included diabetes in the covariates (17). Similarly, alcohol was included in our study as a common variable affecting cognition (19). In order to mitigate any potential confounding bias caused by these factors, the statistical models were adjusted for the covariates to control for their impact Gender (male, female) and race (Mexican American, Non-Hispanic Black, Non-Hispanic White, Other Hispanic, Other Race – Including Multi-Racial); Income status (below poverty line: ≤1, above poverty line: >1) (20); education statue (below high school, More than or equal to high school); Smoking status (yes, no); Alcohol consumption status (yes, no); hypertension was determined by questionnaire and blood pressure measurements. The questionnaire included the following questions: Has your doctor ever told you that you have high blood pressure and are you taking high blood pressure medication? The threshold for hypertension is a systolic blood pressure of >140 or a diastolic blood pressure of >90. Three subjects were diagnosed with hypertension when they met these criteria. Blood pressure (systolic and diastolic) measurements were performed at the MEC (21); Diabetes mellitus was defined as conditions such as reporting of diabetes mellitus diagnosis and use of diabetes medication or insulin; Stroke is categorized as yes or no (22).

### Statistical analysis

For statistical analyses, R Studio (version 4.2.3–2009-2023 RStudio) used to be beforehand used. Baseline traits of the find out about populace have been expressed as percentages in accordance to cognitive impairment sample status. Weighted logistic regression fashions have been used to examine the relationship between cognitive impairment and Tinnitus. Crude Model was unadjusted; model 1 adjusted for demographic element, gender race and PIR; and model 2 adjusted for model 1 and cognitive impairment-related disease high blood pressure, diabetes mellitus and stroke; model 3 adjusted for model 2 and cognitive impairment-related adverse lifestyle smoking, alcohol use. Linear regression analysis was conducted to calculate the *β* coefficients representing the association between cognitive function variables and periodontal status. The model was adjusted using logistic regression. Statistical significance was determined for all results with a *p*-value less than 0.05. Traditional regression approaches select a single “best” model, ignoring uncertainty in variable selection and inflating Type I errors. BMA addresses this by averaging posterior distributions of parameters across all possible models, weighted by their posterior model probabilities. This reduces overfitting and provides robust estimates of variable importance. Analyses were performed in R 4.3.1 using the BMA package, which employs a Metropolis-Hastings algorithm to explore the model space. MCMC Settings: Iterations: 100,000 Markov Chain Monte Carlo (MCMC) steps after 10,000 burn-in. For computational efficiency, Bayesian Information Criterion (BIC) approximations were used to estimate posterior model probabilities.

## Results

Participants with cognitive impairment assessments and relevant examinations were included in the study using data obtained from the NHANES database. Table 1 summarizes the general characteristics of participants with different tinnitus conditions. Continuous variables include age, CERAD-WL score, AFT score, DSST score; categorical variables include gender, race, education level, PIR, smoking status, alcohol consumption status, hypertension, diabetes, and stroke. Except for gender (*p* = 0.76), hypertension (*p* = 0.27), diabetes (*p* = 0.17), and smoking status (*p* = 0.49), all other variables showed statistical significance.

**Table 1:** Population characteristics in NHANES adults with different tinnitus.

Variable	Total	Normal	Acute tinnitus	Non-acute tinnitus	*P-*value
Continuous variables, mean ± SD					
Age	63.88 ± 0.21	63.65 ± 0.20	64.00 ± 1.07	64.58 ± 0.35	**0.02**
CERAD-total	26.76 ± 0.54	26.75 ± 0.52	22.25 ± 1.78	26.99 ± 0.92	**0.02**
AFT	20.17 ± 0.40	20.07 ± 0.43	11.74 ± 1.23	20.80 ± 0.58	**<0.0001**
DSST	58.36 ± 1.01	58.85 ± 1.05	30.04 ± 2.28	58.03 ± 2.32	**<0.0001**
Categorical variables, *N* (%)					
Gender					0.76
Female	345(51.95)	258(51.57)	12(68.04)	75(52.43)	
Male	339(48.05)	269(48.43)	7(31.96)	63(47.57)	
Race					**0.04**
Mexican American	66(3.12)	46(2.81)	6(32.49)	14(2.90)	
Non-Hispanic Black	228(8.47)	184(9.03)	6(22.41)	38(6.28)	
Non-Hispanic White	216(78.36)	161(79.30)	2(27.92)	53(77.55)	
Other Hispanic	98(3.74)	74(3.85)	5(17.18)	19(2.88)	
Other Race – Including Multi-Racial	76(6.31)	62(5.02)	0(0.00)	14(10.39)	
Edu					**0.02**
Below high school	171(12.22)	131(11.26)	11(55.89)	29(13.36)	
More than or equal to high school	513(87.78)	396(88.74)	8(44.11)	109(86.64)	
PIR					**0.02**
<=1	140(9.69)	99(9.33)	6(41.29)	35(9.50)	
>1	544(90.31)	428(90.67)	13(58.71)	103(90.50)	
DM					0.17
No	388(63.34)	302(64.25)	5(17.94)	81(62.42)	
Yes	296(36.66)	225(35.75)	14(82.06)	57(37.58)	
Alcohol.user					**0.04**
No	107(11.19)	85(12.06)	4(34.01)	18(7.70)	
Yes	577(88.81)	442(87.94)	15(65.99)	120(92.30)	

Bolded font indicates *p* < 0.05. N, number of participants; SD, standard deviation. AFT, Animal Fluency Test; DSST, The Dementia SomaSignal Test; PIR, Ratio of family income to poverty; DM, diabetes mellitus.

### Multiple linear regression analyses

Because, there are discrepancies in the results obtained from different regression models, and uncertainty exists regarding the superiority of models under different evaluation criteria, indicating model uncertainty. To address this issue, Bayesian Model Averaging (BMA) can be employed. BMA considers all possible regression models and assigns weights to them, thereby integrating the strengths of different regression models and overcoming model uncertainty. This approach can potentially yield better results in analyzing the relationship between tinnitus and cognitive impairment. So, we use BMA to find which model is best.

## Discussion

In this study, we investigated the relationship between tinnitus status and three cognitive function measures, including CERAD-WL, AFT, and DSST, in a sample of adult participants in the United States. Compared to healthy participants, those with acute tinnitus had lower scores (Table 2). In the regression analysis, we found significant associations between tinnitus and AFT and DSST scores, except for CERAD-WL (Table 3). Even after adjusting for all other factors to minimize potential confounding, tinnitus remained significantly associated with increased AFT and DSST scores in Model 3 (*p*-values of 0.005 for both) (Tables 4, 5).

**Table 2:** Bayesian model averaging of associations between CERAD-total and tinnitus.

Variable	*p*! = 0	EV	SD	Model 1	Model 2	Model 3	Model 4	Model 5
Intercept	100	29.444027	7.05205	35.0769	22.6574	35.9814	35.1917	23.4951
EDU	100	4.185311	0.5206	4.0395	4.1204	4.4289	3.9534	4.5123
Sex	100	−3.130511	0.42127	−3.1259	−3.1264	−3.0425	−3.1051	−3.0426
pir	60.3	0.86325	0.81302	1.4675	1.4751		1.3993	
DM	34.5	−0.331372	0.51764				−0.9145	
Age	52.8	−0.10064	0.10912	−0.1934		−0.1945	−0.1873	
Tinnitus	2.3	0.029987	0.25562					
Alcohol.user	22.5	0.266733	0.56774					
NVar				4	3	3	5	2
*r*^2^				0.194	0.186	0.185	0.2	0.177
BIC				−121.5348	−121.1042	−120.3317	−119.922	−119.9041
Posterior Prob				0.17	0.137	0.093	0.076	0.075

EDU, education; NVar, number of variables; BIC, Bayesian Information Criterion.

BMA analysis of CERAD-Total-tinnitus associations demonstrated: Strong predictors: Education (*β* = 4.19) and female sex (*β* = −3.13) (*p* ≠ 0 = 100%). Sex difference emerged as a novel strong predictor.

**Table 3:** Bayesian model averaging of associations between AFT and tinnitus.

Variable	p! = 0	EV	SD	Model 1	Model 2	Model 3	Model 4	Model 5
Intercept	100	13.4605	0.8765	12.7905	12.5728	13.8933	13.6851	13.0749
Black	20.4	−0.2358	0.5379					
White	100	4.0746	0.5752	4.1915	4.2472	4.3213	4.379	4.1571
Other.Race	20.5	−0.32	0.7312					
Edu	100	3.0664	0.4845	2.987	3.1234	3.0361	3.1746	3.0371
Less.than.three.months	51.6	−1.6049	1.7799	−3.0671		−3.1002		
pir1.1	13.3	0.1254	0.3704					
Alcohol.user	49.8	0.6886	0.7901	1.3984	1.4133			1.3976
DM	31	−0.2772	0.4695					−0.9142
nVar				4	3	3	2	4
*r*^2^				0.231	0.223	0.223	0.215	0.229
BIC				−154.2126	−154.114	−153.0083	−14152.8348	−151.9336
post prob				0.11	0.104	0.099	0.091	0.058

EDU, education; NVar, number of variables; BIC, Bayesian Information Criterion.

BMA analysis of AFT-tinnitus associations identified: Strong predictors: White race (*β* = 4.07) and education (*β* = 3.07) (*p* ≠ 0 = 100%). Marginal signals: Short-term exposure (*β* = −1.60, *p* ≠ 0 = 51.6%); Alcohol use (*β* = 0.69, *p* ≠ 0 = 49.8%).

**Table 4:** Bayesian model averaging of associations between DSST and tinnitus.

Variable	*p*! = 0	EV	SD	Model 1	Model 2	Model 3	Model 4	Model 5
Intercept	100	13.4605	0.8765	12.7905	12.5728	13.8933	13.6851	13.0749
Black	20.4	−0.2358	0.5379					
White	100	4.0746	0.5752	4.1915	4.2472	4.3213	4.379	4.1571
Other.Race	20.5	−0.32	0.7312					
Edu	100	3.0664	0.4845	2.987	3.1234	3.0361	3.1746	3.0371
Less.than.three.months	51.6	−1.6049	1.7799	−3.0671		−3.1002		
pir1.1	13.3	0.1254	0.3704					
Alcohol.user	49.8	0.6886	0.7901	1.3984	1.4133			1.3976
DM	31	−0.2772	0.4695					−0.9142
nVar				4	3	3	2	4
*r*^2^				0.231	0.223	0.223	0.215	0.229
BIC				−153.2126	−153.114	−153.0083	−14152.8348	−151.9336
Post prob				0.11	0.104	0.099	0.091	0.058

EDU, education; NVar, number of variables; BIC, Bayesian Information Criterion.

BMA analysis of DSST-tinnitus associations showed the same result with BMA analysis of AFT-tinnitus associations acute tinnitus (*p* < 0.0001), and this relationship remained unchanged after adjusting for models 1, 2, and 3. However, the risk of cognitive impairments in non-acute tinnitus patients is unclear, as shown in Table 5.

**Table 5:** Associations between cognitive impairment and tinnitus.

Model	None	Acute tinnitus	*P*	Non-acute tinnitus	*P*
Crude model	ref	−28.81(−33.92,-23.70)	<0.0001	−0.82(−6.31, 4.68)	0.76
Model 1	ref	−17.14(−22.94,-11.34)	<0.0001	−1.12(−6.46, 4.21)	0.65
Model 2	ref	−15.26(−20.66,-9.87)	<0.001	−1.03(−5.98, 3.92)	0.64
Model 3	ref	−16.47(−21.98,-10.96)	<0.001	−1.32(−6.80, 4.17)	0.60

Crudel model: None.

Model 1: sex, eth, pir.

Model 2: sex, eth, pir, DM.

Model 3: sex, eth, pir1, DM, alcohol.user.

According to a report by the Alzheimer’s Association in 2015, the biggest danger element for Alzheimer’s disorder and different dementias is age (23). Additionally, other reports have indicated a close relationship between tinnitus and age. Moreover, there have been other reports suggesting an inseparable connection between tinnitus and age (9). There is strong evidence to suggest that smoking and excessive alcohol consumption are risk factors for dementia (9). Although the evidence level is limited, conditions such as hypertension, diabetes, and stroke may still be risk factors for cognitive impairment (9). This large-scale study allows us to adjust for various modifiable risk factors using different models to achieve optimal results.

Cognitive function in the NHANES database was assessed using CERAD-WL, AFT, and DSST.CERAD-WL evaluates the immediate and delayed learning ability of new verbal information (24, 25); AFT is used to verify govt feature as properly as different functions, such as semantic reminiscence and processing velocity (26); DSST serves as a international measure of intelligence health, relying on processing speed, visible scanning, sustained attention, and momentary reminiscence (27). In our study, although CERAD-W scores indicated no association between tinnitus and cognitive function (*β* = −1.56, 95% CI = −6.63, 3.52), AFT and DSST were significantly correlated with tinnitus (*p* = 0.02, *p* = 0.005). Participants with acute tinnitus exhibited poorer AFT and DSST scores in fully adjusted Model 3 in Tables 4, 5 (*β* = −4.44, 95% CI = −7.78, −1.10; *β* = −14.75, 95% CI = −21.88, −7.62). This intriguing phenomenon may suggest that the hypothesized negative impact of tinnitus on cognitive impairment is more related to cognitive impairments in attention, memory, and executive function, while CERAD-WL may focus more on language abilities. Additionally, our assessment of CERAD-WL is based on the total score, which may lead to some limitations in evaluating immediate and delayed learning abilities. Logistic regression revealed that tinnitus was a risk factor for cognitive impairment (OR: 23.10; 95% CI = 8.74, 61.08), and this association remained significant in the fully adjusted model (OR: 5.28; 95% CI = 1.12, 24.86).

In recent years, there has been a proliferation of research on cognitive impairment, and the evidence linking tinnitus to cognitive impairment has attracted global researchers’ attention. A study from China indicated that patients with severe tinnitus (THI ≥ 38) demonstrated noticeable cognitive impairments when assessed using a cognitive ability screening tool. Furthermore, there is a strong correlation between the severity of tinnitus and the degree of cognitive impairment (28). Interestingly, Lee et al. demonstrated through a cross-sectional study that tinnitus is a potential independent determinant for predicting mild cognitive impairment (29). Research on tinnitus-related cognitive impairments is commonly guided by means of one of two hypotheses. The “general useful resource depletion” speculation suggests that the understanding of tinnitus leads to sustained orientation to sound, thereby decreasing normal processing capacity (30). The “controlled processing” speculation posits extra particular results of tinnitus, suggesting that sufferers must be in a position to function easy duties at stages comparable to a non-tinnitus manage group, however overall performance can also be affected in greater hard duties and multitask paradigms (30). Lavie et al. (31) proposed that when there is excessive attention to a specific aspect, other functions in the brain may be suppressed, and the perception of tinnitus can lead to sustained orientation to sound, thereby reducing overall processing capacity. Tegg-Quinn et al. (11) suggest that tinnitus may affect attentional control and executive control in cognitive abilities, resulting in difficulties in attention, distraction, and reduced information processing.

Currently, there is a theory that explains the impact of tinnitus on cognitive performance, which is gradually gaining widespread attention. Khan and Husain proposed the load theory to explain the relationship between tinnitus and cognition (30). The load theory is a framework that addresses the cognitive and neurobiological aspects, explaining the capacity of human attentional resource management and the limitations of cognitive load. The fundamental concept of this theory is that limited attentional resources should not be overloaded during task execution, as overload can lead to resource dispersion and interference, thus affecting task performance. From the perspective of the load theory, the assumption mentioned above suggests that the controlled processing hypothesis of tinnitus implies cognitive resource overload, resulting in decreased capacity and poorer performance in tasks involving higher-order cognitive functions (e.g., working memory). The everyday depletion speculation suggests that tinnitus consumes perceptual and cognitive sources – if overall performance is affected in all interest and cognitive manage tests, it is feasible that perceptual sources are thoroughly occupied, ensuing in poorer overall performance in detection tasks, and cognitive assets are additionally affected. Overload leads to a decline in performance in discriminating tasks (32). Resting-state purposeful magnetic resonance imaging displays extended neural endeavor and altered neuronal exercise in tinnitus sufferers in contrast to wholesome individuals, indicating feasible perceptual aid overload in the course of tinnitus (33). Additionally, research suggests that cholinergic dysfunction may be a primary cause of age-related tinnitus associated with presbycusis and age-related cognitive impairment. The loss of cholinergic neural innervation consequences in lowered cholinergic anti-inflammatory results and decreased neuroglial activation, main to the loss of GABAergic interneurons. Therefore, this could be another key step in the pathological mechanisms of tinnitus and cognitive impairment (34).

Another interesting finding of this study is that there is no significant association between non-acute-tinnitus and cognitive function, which differs from previous research (35). This could be due to the higher tolerance of chronic tinnitus patients toward their tinnitus, resulting in less cognitive resources being occupied by tinnitus. Therefore, the association between non-acute tinnitus and cognitive function is less apparent (32). Another possible explanation is that the brain regions activated by acute and non-acute tinnitus are different. Compared to the chronic tinnitus group, the acute tinnitus group showed significantly increased activity in the bilateral superior frontal gyrus and medial prefrontal cortex. On the other hand, the non-acute tinnitus group exhibited increased activity in the left hippocampus, left amygdala, and left temporal pole: superior temporal gyrus. However, there were hyperfunctional connections between the auditory network, cerebellum, reticular formation, amygdala, and hippocampus, which may contribute to the storage or retrieval of emotions, movement, conscious awareness, and tinnitus memory, benefiting the cognition of non-acute tinnitus patients. Additionally, the superior frontal gyrus is primarily involved in emotion regulation, which may consume more perceptual resources, leading to an overload state that favors the development of cognitive impairments (36). By reconciling conflicting literature and candidly addressing limitations, this study advances a nuanced perspective: non-acute tinnitus may signal or contribute to cognitive vulnerability in aging populations, but its role is likely contingent on context, duration, and comorbidities. Future research must prioritize mechanistic clarity and translational relevance to guide clinical practice.

## Conclusion

Individuals with tinnitus exhibited significantly lower scores in AFT and DSST compared to those without tinnitus. Moreover, there was a significant correlation between tinnitus and impaired cognitive function, consistent with previous research, indicating an association between tinnitus and cognitive impairments.

### Shortage

Given the limitations of this study, further research is needed, including mechanistic experiments and longitudinal studies, to explore the underlying mechanisms behind this specific correlation and understand the direct impact of tinnitus on cognitive impairments. Moreover, due to the small pattern size, this learn about did now not behavior similarly evaluation and exploration of continual tinnitus, which may also end result in some biased results. The small acute tinnitus cohort limited statistical power to detect modest effects, necessitating replication in targeted cohorts. Tinnitus chronicity is a continuum, and a six-month threshold might better distinguish transient vs. persistent cases in some contexts. However, NHANES data granularity precluded finer stratification. Self-reported duration in NHANES may introduce inaccuracies, as participants might misremember the onset or severity of symptoms.

Emerging evidence suggests that tinnitus chronicity, even in the absence of detailed subtyping, independently predicts cognitive burden. For instance, chronic tinnitus (>3 months) has been linked to maladaptive neuroplasticity and sustained cognitive load, whereas acute cases often resolve spontaneously. Our classification thus targets a clinically meaningful transition point in the tinnitus trajectory.

While we fully agree that tinnitus heterogeneity (e.g., severity, laterality, comorbidities) is critical, the NHANES dataset imposes constraints, but these limitations preclude the application of contemporary subtyping methodologies.

While these current classification approaches are methodologically superior, they require rich, multidimensional datasets unavailable in NHANES. Our study instead prioritizes population-level generalizability, complementing hypothesis-generating clinical studies.

The acute tinnitus group’s small sample size limits our ability to detect subtle cognitive effects. However, the consistency of point estimates (e.g., negative trends in AFT/DSST) with the non-acute group suggests plausible biological continuity, warranting further investigation in larger cohorts. The temporal sequence between tinnitus and cognitive decline cannot be established. Bidirectional relationships are plausible—chronic tinnitus may exacerbate cognitive load through maladaptive neuroplasticity, while preexisting cognitive deficits could impair coping mechanisms, amplifying tinnitus perception. Longitudinal studies are needed to disentangle causation.

## Data Availability

The datasets presented in this study can be found in online repositories. The NHANES survey can be found: https://www.cdc.gov/nchs/nhanes/?CDC_AAref_Val=https://www.cdc.gov/nchs/nhanes/index.htm.
